# Predictive Role of Lymph Node Germinal Centers in Postoperative Recurrence of Non-Small Cell Lung Cancer Treated With Immune Checkpoint Inhibitor Therapy

**DOI:** 10.7759/cureus.79521

**Published:** 2025-02-23

**Authors:** Yohei Kawaguchi, Munehide Nakatsugawa, Nanako Nishioka, Taiyo Nakamura, Kentaro Imai, Takuya Aoki, Naohiro Kajiwara, Norihiko Ikeda

**Affiliations:** 1 Department of Thoracic Surgery, Tokyo Medical University Hachioji Medical Center, Hachioji, JPN; 2 Department of Diagnostic Pathology, Tokyo Medical University Hachioji Medical Center, Hachioji, JPN; 3 Department of Pharmacology, Tokyo Medical University Hachioji Medical Center, Hachioji, JPN; 4 Department of Clinical Oncology, Tokyo Medical University Hachioji Medical center, Hachioji, JPN; 5 Department of Surgery, Tokyo Medical University, Shinjuku, JPN

**Keywords:** germinal center, immune checkpoint inhibitors (icis), lymph node dissection, metastatic non-small cell lung cancer, non-small cell lung cancer (nsclc)

## Abstract

Introduction

The germinal center (GC) in lymph nodes plays an important role in immune responses; however, their relevance to the effects of immune checkpoint inhibitor (ICI) remains unclear. The relationship between GC and ICI efficacy is investigated in this study.

Materials and methods

This investigation included 16 non-small cell lung cancer (NSCLC) patients with postoperative recurrence who were treated with immune checkpoint inhibitors (ICI) between January 2016 and April 2022. Patients were categorized into two groups based on the presence of GC in the lymph nodes. Additionally, the association between the number of lymph nodes dissected during pulmonary resection and ICI efficacy was evaluated.

Results

Sixteen patients were included with eight GC+ patients and eight GC- patients. The presence of GC positively influenced ICI efficacy, with the objective response rate (ORR) being significantly higher in the GC+ group (62.5%) compared to the GC- group (12.5%) (p=0.039). Disease control rate (DCR) was also more favorable in the GC+ group (100%) compared to the GC- group (50%) (p=0.021). Additionally, patients with fewer lymph nodes dissected at surgery had a better progression-free survival (median: 15.7 months) than those with more lymph nodes dissected (median: 7.4 months) (p=0.027).

Conclusion

GC in the lymph nodes can enhance the efficacy of ICI in treating NSCLC. Moreover, the number of dissected lymph nodes has emerged as a crucial prognostic factor that influences the effectiveness of treatment. These findings underscore the importance of considering lymph node characteristics in personalized ICI therapy for NSCLC.

## Introduction

The treatment strategy for non-small cell lung cancer (NSCLC) has evolved significantly with the introduction of immune checkpoint inhibitors (ICIs). These agents, which target the PD-1 and PD-L1 pathways, have shown promising results in enhancing the ability of the immune system to recognize and eliminate cancer cells [1]. Initially authorized for advanced, inoperable NSCLC patients, ICIs have subsequently broadened their scope to early-stage, operable NSCLC [2-4]. Thus, it is imperative to enhance the predictive accuracy of ICIs by using biomarkers. The tumor microenvironment (TME) has gained prominence as a vital area of investigation in this context, as it is a complex milieu of various cellular and non-cellular components that interact dynamically to influence cancer progression and treatment outcomes [5]. Tertiary lymphoid structures (TLS) have attracted considerable attention in the TME because of their probable involvement in modifying the efficacy of ICI. TLS are ectopic lymphoid formations that resemble secondary lymphoid organs and have been shown to be involved in antitumor immunity [6-8]. Despite the increasing recognition of TLS in cancer treatment, there is a significant knowledge gap concerning the specific elements within the TME that may be pivotal for modulating ICI outcomes. One such element is the germinal center (GC).

GC are specialized anatomical compartments located within secondary lymphoid organs, such as lymph nodes, and are dedicated to B-cell maturation and the generation of antibodies [9]. Increasing research indicates that GC has an impact on the maturation of TLS. The presence of follicular dendritic cells within the germinal center has been observed to improve antigen presentation, potentially augmenting the efficacy of immune checkpoint inhibitors [10,11]. However, the relationship between GC and ICI treatment outcomes remains unclear. In terms of lymph nodes, changes in the effects of ICI with lymph node dissection have also been reported. The tumor-draining lymph node (TDLN) is the initial site of immune activation in NSCLC [12]. ICI efficacy has been observed to diminish in mice in which the TDLN was resected from head and neck squamous cell carcinoma [13]. This result suggests that surgical lymph node dissection carries the potential risk of compromising ICI efficacy. From these reports, it can be inferred that lymph nodes and GC play an important role in the immune response to ICI. Therefore, we searched for GC in dissected lymph nodes and examined the effects of GC and TME on ICI efficacy.

## Materials and methods

Study design and patient selection

**Figure 1:**
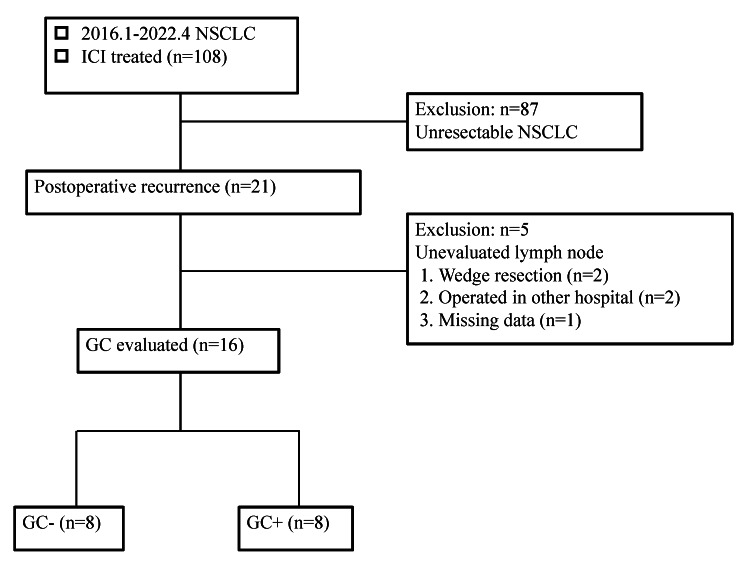
Flow chart of the study population. Of the 21 patients with postoperative recurrence, 16 patients whose lymph nodes could be evaluated were divided into two groups according to the presence or absence of GC, respectively. GC: germinal center; NSCLC: non-small cell lung cancer; ICI: immune checkpoint inhibitor

This study was a retrospective analysis of NSCLC patients with postoperative recurrence who were treated with ICI between January 2016 and April 2022 at Tokyo Medical University Hachioji Medical Center. During the study period, 108 patients underwent ICI treatment. Among these patients, 21 who experienced postoperative recurrence were considered for inclusion in the study. Tissue specimens from these patients were stored at Tokyo Medical University Hachioji Medical Center, and the same tissue was collected from the hospital for this study. Patients with unresectable NSCLC who had not undergone surgery were excluded because their lymph nodes could not be evaluated. Patients with postoperative recurrence in this study were defined as patients with primary lung cancer who had undergone curative resection and had imaging or pathologic evidence of recurrent disease. Furthermore, the following four patients were excluded: two who underwent wedge resection, which made lymph node evaluation impossible, two who received treatment at another hospital, and one with missing data. The remaining 16 patients for whom pathological assessment of GC was feasible were included in the final analysis (Figure 1). 

Data collection

Clinicopathological variables and patient backgrounds were retrieved from the medical records. Immunohistochemical expression of PD-L1 was evaluated using the tumor proportion score (TPS) of the resected tumor, and categorized into three groups as follows: <1%, 1-49%, and ≥50%. TPS was evaluated through immunohistochemistry using the 22C3 pharmDx test (Carpinteria, CA: Dako North America). The indications for ICI in patients with lung cancer were determined through collaborative discussions at the Cancer Board of Tokyo Medical University Hachioji Medical Center. The patients were selected based on the availability of medical records and evaluable lymph node samples. All patients underwent curative surgical resection and had a confirmed diagnosis according to the TNM Classification for Lung and Pleural Tumors of the Union for International Cancer Control (TNM Eighth Edition) [14].

Ethical statement

Ethical approval was obtained from the Tokyo Medical University Medical Ethics Review Board (approval number: T2023-0074). The requirement for written informed consent was waived in accordance with the study protocol [15].

Pathological evaluation

**Figure 2:**
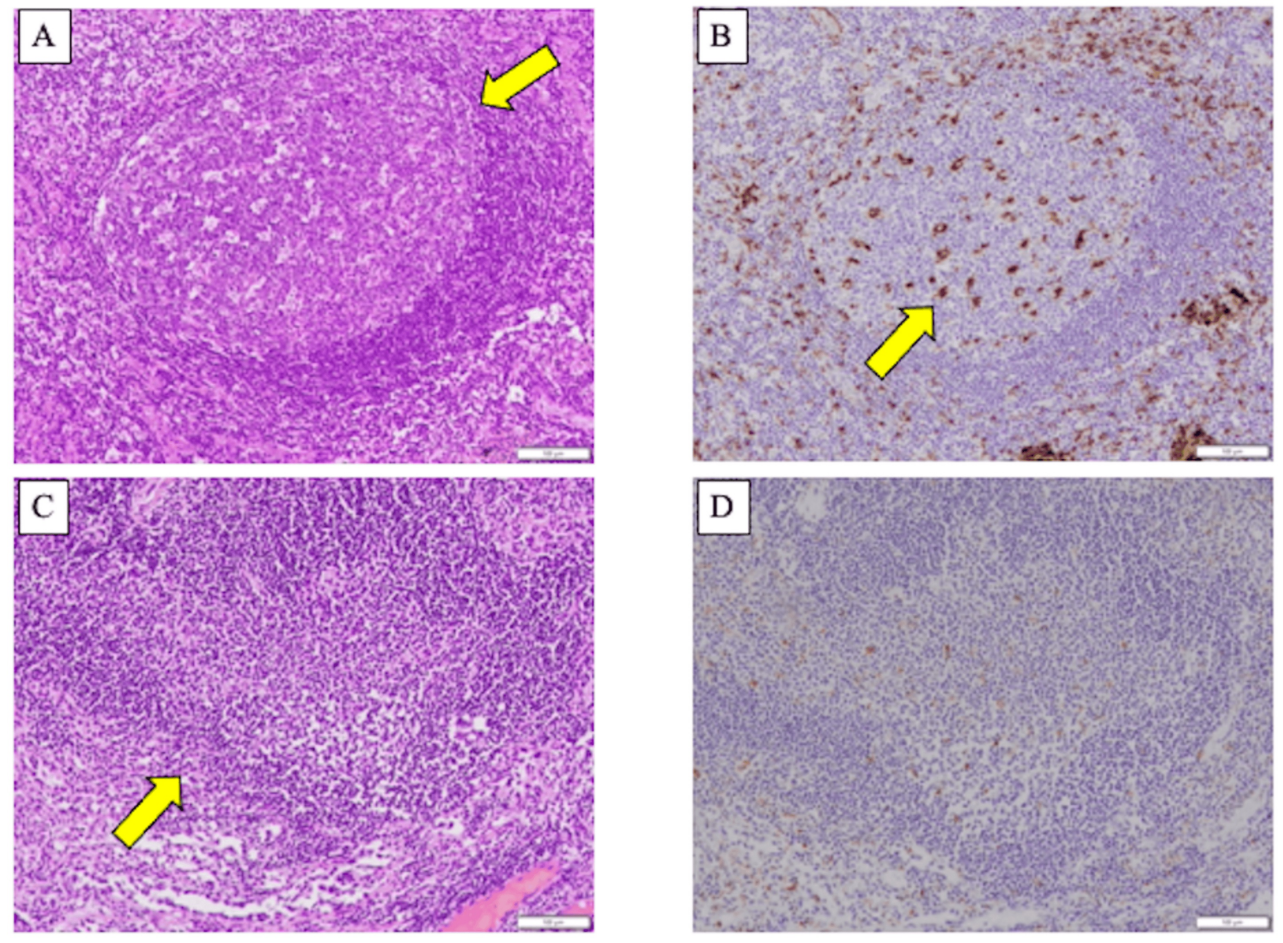
Pathological images of germinal center. (A and C) Germinal centers (GC) in lymph node section of a patient with non-small cell lung cancer, stained with hematoxylin and eosin (40x magnification in A and 200x magnification in C). (B and D) The same section was subjected to immunohistochemistry using antibodies against CD68 (200x magnification in B and D). (A and C) Clear follicular structure of the lymph node germinal centers (arrow). (B) Macrophages stained with CD68, a marker specific to these cells (arrow).

Lymph nodes were assessed using surgically dissected mediastinal or hilar lymph nodes. All the lymph node samples were fixed in 10% formalin and embedded in paraffin. Hematoxylin and eosin (HE) staining was performed for histological analysis. Patients were categorized into the following two groups: GC+ (presence of GC) and GC- (absence of GC). GC was identified on HE stained slides by the presence of a well-defined follicle, with a follicular center containing a mixture of immunoblasts, lymphocytes, and macrophages [12]. In this study, we defined GC as distinct follicular structures identified by hematoxylin and eosin (HE) staining and positive staining for CD68. Immunobiological staining with CD68, which is highly expressed in macrophages (KP1, DAKO, M0814), was also performed to confirm GC. In the GC+ group, well-defined follicular structures were observed (Figure 2A), which stained positively for CD68 (Figure 2B). Conversely, in the GC- group, the boundaries of the follicular structures were indistinct (Figure 2C) and CD68 staining was negative (Figure 2D). GC evaluation was performed by an experienced pathologist (MN). The number of dissected lymph nodes was counted by the same experienced pathologist (MN). All resected lymph nodes were evaluated, and cases were classified as GC+ if at least one lymph node contained a GC. In this study, we chose to evaluate GCs in non-metastatic lymph nodes because lymph node metastasis has been reported to influence the TME, potentially altering immune responses within the affected nodes [16].

Clinical outcomes

The endpoints of this study were the ICI response and survival outcomes. We evaluated the efficacy of ICI by calculating the objective response rate (ORR), which is the sum of complete response (CR) + partial response (PR), and the disease control rate (DCR), which is the sum of CR + PR + stable disease (SD). Radiological imaging includes chest radiography, chest or abdominal computed tomography (CT), brain magnetic resonance imaging (MRI), and positron emission computed tomography (PET-CT). ICI effectiveness was evaluated in accordance with the Response Evaluation Criteria in Solid Tumors (RECIST) v1.1 principle [17]. The tumor shrinkage rate was measured as the change in the long diameter multiplied by the short diameter of the tumor at its widest plane of dissection.

We examined progression-free survival (PFS) as the survival outcome. PFS was defined as the time elapsed from the date of the first ICI administration to the date of progression or death. We tested whether the presence or absence of GC and the number of dissected lymph nodes affected the effectiveness of ICI.

Statistical analysis

Continuous variables were compared using the Mann-Whitney U test and categorical variables were compared using Fisher’s exact test. The ORR and DCR between the GC+ and GC- groups were compared using the chi-squared test. Kaplan-Meier survival curves were generated to assess PFS, and the log-rank test was used to compare the survival distributions. When drawing survival curves based on the number of lymph nodes dissected, ROC curves were created, and cutoff values were calculated to divide the patients into two groups. Statistical significance was set at p<0.05. All statistical analyses were performed using the SPSS statistical software package version 29.0 (Chicago, IL: SPSS Inc.).

## Results

Patient characteristics

Sixteen patients with postoperative recurrent NSCLC were included in the study. Patient characteristics are shown in Table 1. The median age of the patients was 71 (range: 63-85) years. All the patients underwent lobectomy. The most commonly used ICI was pembrolizumab (11 of 16 patients). Of these, eight patients were categorized as GC+ and eight as GC-. The median number of dissected lymph nodes was 14.5 in GC+ and 14 in GC-. In the GC+ group, 25% of patients had TPS ≥50%; and in the GC- group, 25% of patients had TPS ≥50%. The median number of dissected lymph nodes was 14.5 in the GC+ group and 14 in the GC- group.

**Table 1:** Summary of patient clinicopathological characteristics. GC: germinal center; ICI: immune checkpoint inhibitor; LND: lymph node dissection; TPS: tumor proportion score; CPS: combined positive cell score; IC: immune cell score

Variables		GC+ (n=8) (%)	GC- (n=8) (%)	p-Value
Sex	Male	4 (50)	6 (75)	0.302
	Female	4 (50)	2 (25)	
Age (years)	≥75	6 (75)	6 (75)	1.000
	<75	2 (25)	2 (25)	
Pack year	<10	6 (75)	6 (75)	1.000
	≥10	2 (25)	2 (25)	
Pathological stage	I	3 (38)	4 (50)	0.788
	II	2 (25)	1(12)	
	III	3 (38)	3 (38)	
Histology	Adenocarcinoma	3 (37)	7 (88)	0.111
	Squamous cell carcinoma	4 (50)	1 (12)	
	Other	1 (12)	0	
Lymph node dissection	Selective	2 (25)	1 (12)	0.522
	Systematic	6 (75)	7 (88)	
Number of LND	≥17	5 (63)	1 (12)	0.039
	<17	3 (37)	7 (88)	
Recurrence site	Local	2 (25)	1 (12)	0.522
	Distant	6 (75)	7 (88)	
Treatment type	Chemotherapy + ICI	5 (63)	4 (50)	0.614
	ICI alone	3 (37)	4 (50)	
ICI regimen	Pembrolizumab	5 (63)	6 (76)	0.489
	Nivolumab ± ipilimumab	3 (37)	1 (12)	
	Atezolizumab	0	1 (12)	
Treatment line	1st line	6 (75)	6 (75)	1.000
	Late line	2 (25)	2 (25)	
TPS	<1% or unknown	4 (50)	3 (37)	0.842
	1-49%	2 (25)	3 (37)	
	≥50%	2 (25)	2 (25)	
CPS	<1% or unknown	2 (25)	1 (12)	0.065
	1-49%	0	4 (50)	
	≥50%	6 (75)	3 (38)	
IC	IC1 or unknown	2 (25)	3 (38)	0.626
	IC2	0	1 (12)	
	IC3	6 (75)	4 (50)	
CA9	Presence	2 (25)	2 (25)	1.000
	Absence	6 (75)	6 (75)	

Efficacy of ICI

**Figure 3:**
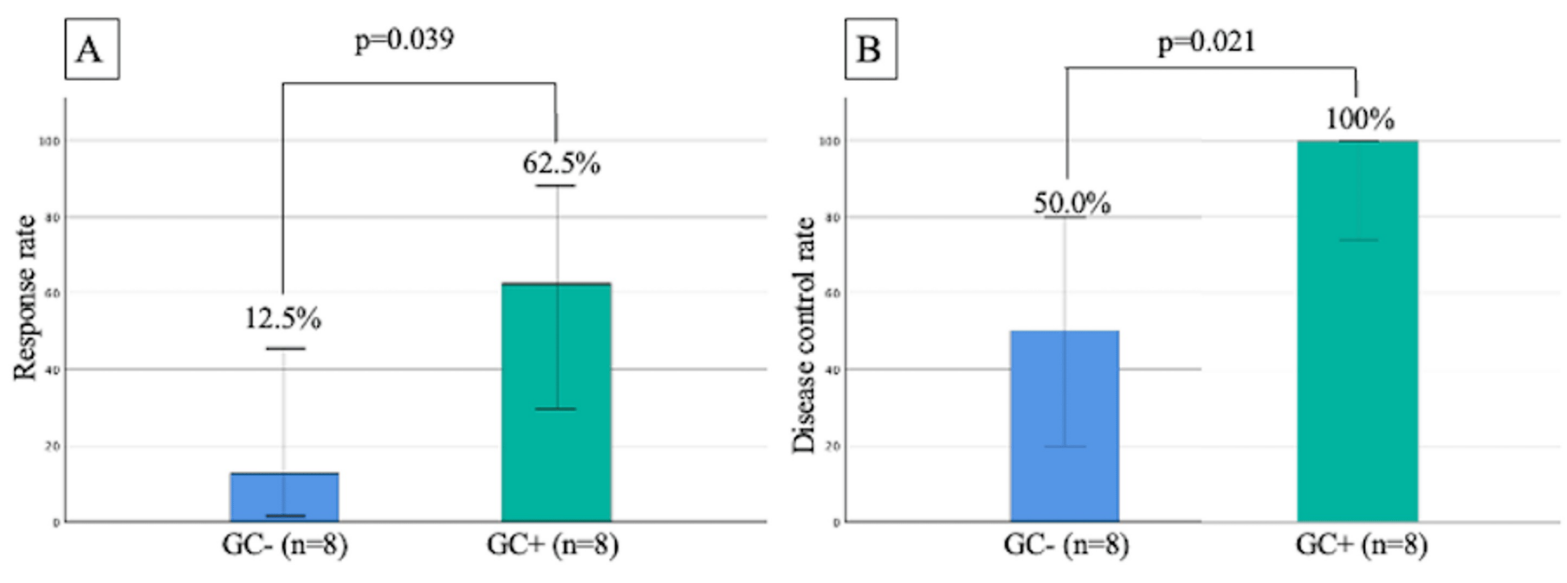
The difference in the best overall response of ICI with or without GC tested by chi-square test. The images show (A) objective response rate and (B) disease control rate. GC: germinal center; ICI: immune checkpoint inhibitor

**Figure 4:**
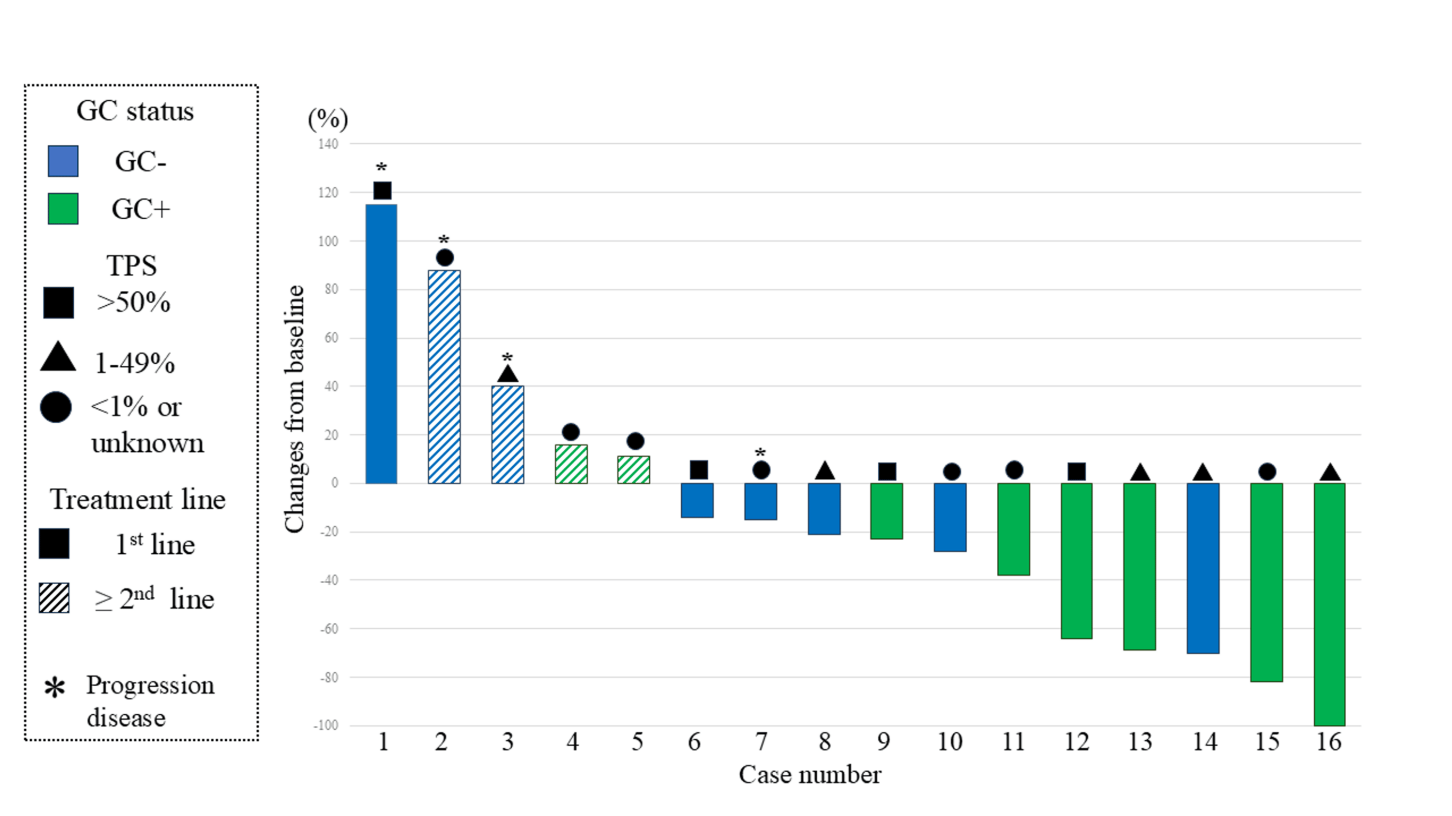
Waterfall plot showing the rate of tumor shrinkage at the time of best overall response according to GC. Progression of disease refers to disease progression as defined by the Response Evaluation Criteria in Solid Tumors (RECIST). GC: germinal center; TPS: tumor proportion score

Differences in the efficacy of ICI according to GC are shown in Figures 3A, 3B. The ORR was significantly higher in the GC+ group than that in the GC- group. Specifically, five patients (62.5%) in the GC+ group showed PR compared to one patient (12.5%) in the GC- group (p=0.039) (Figure 3A). In terms of DCR, all eight patients (100%) in the GC+ group achieved disease control, including PR and SD. In contrast, four of eight patients (50.0%) in the GC- group achieved disease control. The GC+ group showed a favorable DCR rate compared with the GC- group (p=0.021) (Figure 3B). The waterfall plot of the change in the tumor size of measurable lesions before and after ICI use is shown in Figure 4.

Most of the patients who achieved tumor reduction were GC+; in particular, two patients who achieved a deep response with shrinkage rates greater than 80% were GC+. Table 2 shows the effect of ICI and the details of patient backgrounds of each patient. Of the six patients who achieved PR or CR, two had local recurrence, and GC+ patients who achieved PR included those with distant metastatic recurrence, such as CNS recurrence. In the GC- group, none of the cases with unknown or <1% TPS achieved PR, whereas in the GC+ group, one out of four (25%) cases achieved PR.

**Table 2:** Association between presence of lymph node germinal centers and treatment response. GC: germinal center; PUL: pulmonary metastasis; LYM: lymph node; ADR: adrenal glands; BRA: brain; HEP: liver; PLE: pleurisy; OSS: bone; CR: complete response; PR: partial response; SD: stable disease; PD: progression disease; TPS: tumor proportion score; LND: lymph node dissection; ICI: immune checkpoint inhibitor; Nivo: nivolumab; Ipi: ipilimumab; Pem: pembrolizumab; Atezo: atezolizumab

S. no.	GC	Pathological stage	TPS	Number of LND	Treatment line	Recurrence site	ICI	Objective response
1	-	I	Unknown	14	3	PUL, LYM	Nivo	PD
2	+	I	Unknown	40	3	LYM, ADR	Nivo	SD
3	+	III	<1%	29	4	PUL, LYM, Pleural effusion	Nivo	SD
4	+	II	<1%	16	1	PUL	Nivo + Ipi	PR
5	-	I	≥50%	24	1	BRA	Pem	PD
6	+	II	≥50%	17	1	BRA, Meningeal dissemination	Pem	SD
7	+	III	Unknown	31	1	PUL, HEP, ADR	Pem	PR
8	+	I	1-49	12	1	LYM	Pem	CR
9	-	I	1-49	16	4	PUL, HEP, PLE	Pem	PD
10	-	III	≥50%	8	1	LYM, OSS	Pem	SD
11	+	III	1-49	11	1	LYM	Pem	PR
12	+	I	≥50%	18	1	PUL, LYM, PLE	Pem	PR
13	-	II	<1%	11	1	PLE	Pem	PD
14	-	I	1-49	2	1	LYM	Pem	PR
15	-	II	1-49	15	1	PUL, LYM	Pem	SD
16	-	III	Unknown	14	1	LYM	Atezo	SD

Survival outcomes

**Figure 5:**
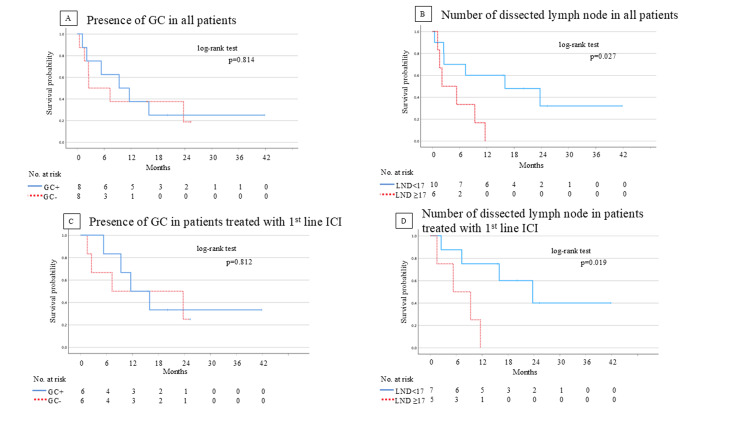
Kaplan-Meier curves for PFS of ICI according to GC and the number of dissected lymph nodes in all patients (A, B) and in patients treated with ICI as first-line therapy (C, D). PFS: progression-free survival; GC: germinal center; TPS: tumor proportion score; LND: lymph node dissection; ICI: immune checkpoint inhibitor

The Kaplan-Meier curve in relation to GC was demonstrated. The median PFS was 10.1 months for the GC+ group and 4.9 months for the GC- group. Although the difference was not statistically significant (p=0.814), a tendency towards improved PFS was observed in the GC+ group (Figure 5A). The AUC value for recurrence or death based on the number of dissected lymph nodes was 0.604 and the optimal cut-off value was 16.5. The Kaplan-Meier curve in relation to the number of dissected lymph nodes is illustrated in Figure 5B. The median PFS was 15.7 months for patients with fewer than 17 lymph nodes removed and 7.4 months for those with 17 or more lymph nodes removed. Patients with fewer than 17 lymph nodes removed exhibited significantly better one-year PFS rates of 60.0% compared with 0% in those with 17 or more lymph nodes removed (p=0.027) (Figure 5B). Furthermore, we performed a prognostic analysis stratified by the presence of GC and the number of dissected lymph nodes, focusing only on patients who received ICI as first-line therapy. Among the study population, 12 patients (75%) received ICI as a first-line treatment. The results in this subgroup were consistent with those observed in the entire cohort. Patients with GC showed a trend toward better prognosis (p=0.812) (Figure 5C). Additionally, patients with ≤17 dissected lymph nodes had significantly better survival outcomes compared to those with >17 dissected lymph nodes (p=0.019) (Figure 5D).

## Discussion

This study revealed two noteworthy findings. First, our results suggest that the presence or absence of GC in lymph nodes may significantly impact the efficacy of ICI in the treatment of recurrent NSCLC. Second, the number of lymph nodes dissected during surgery may serve as a valuable prognostic factor, potentially affecting the efficacy of ICI treatment. Our study underscores the pivotal role of GC in lymph nodes as a potential biomarker for the efficacy of ICI in treating NSCLC. This aligns with emerging research that highlights the significance of TME in modulating immune responses. For instance, a study on metastatic melanoma found that human leukocyte antigen-DR, a positive predictive marker of ICI, has an internal GC-like structure and elicits antitumor immunity [18]. Another study on endometrial cancer revealed that TLS, including GC, is associated with favorable survival outcomes and may represent an active immune reaction in the TME [19]. Liu et al. reported that there was a positive correlation between the number of GC formations in cancer-free lymph nodes and good prognosis in triple-negative breast cancer [20]. Our results add to the growing body of evidence demonstrating the critical function of the TME in cancer immunotherapy, particularly GC, and highlight the importance of assessing the presence of GC in lymph nodes as a qualitative parameter for evaluating treatment effects during ICI.

Our second key finding highlights the prognostic value of the number of lymph nodes dissected during surgery in determining the efficacy of ICI treatment for recurrent NSCLC. This finding aligns with the extant body of research that underscores the significance of lymph node dissection in the management of cancer. A previous study revealed that the number of metastatic lymph nodes and zones had a significant predictive value [21]. A clinical trial examining the administration of ICI in melanoma before or following surgery reported improved outcomes when ICI was administered preoperatively, preceding the excision of the primary tumor or lymph nodes [22].

The removal of "healthy" lymph nodes could potentially impact the immune response and efficacy of ICI. Our findings support the significance of the number of dissected lymph nodes as a prognostic factor in the treatment of recurrent NSCLC with ICI, highlighting the need for further investigation into optimized treatment strategies that consider the interplay between surgical intervention and immunotherapy.

The clinical implications of our study have the potential to substantially impact both current practice and future research about the treatment of recurrent NSCLC. Identification of GC in lymph nodes as a potential biomarker for ICI efficacy offers a new avenue for personalized treatment strategies. These insights could serve as a foundation for developing targeted surgical and immunotherapeutic interventions to enhance patient outcomes.

Although this study makes a significant contribution to the field, it is essential to acknowledge its limitations. First, the sample size was relatively small, which may have affected the generalizability of our findings. Second, we did not control for potential confounding variables such as patient age, comorbidities, or previous treatments. Further large-scale studies are required to confirm this hypothesis. Finally, lymph node assessment was performed by a single pathologist, thus lacking blinding and a multidisciplinary review. Despite these limitations, the strength of this study lies in its novel focus on GC and lymph node dissection, which provides a robust foundation for future research. In conclusion, our results suggest that GC inside the lymph nodes may play an important role in the effect of ICI on the postoperative recurrence of lung cancer.

The removal of "healthy" lymph nodes could potentially impact the immune response and efficacy of ICI. Our findings support the significance of the number of dissected lymph nodes as a prognostic factor in the treatment of recurrent NSCLC with ICI, highlighting the need for further investigation into optimized treatment strategies that consider the interplay between surgical intervention and immunotherapy. On the other hand, the number of dissected lymph nodes was lower in the GC- group compared to the GC+ group and the GC- group showed inferior response to ICIs. This finding suggests that in addition to the physical removal of lymph nodes, the quality of the lymph nodes themselves may influence the efficacy of ICIs. Our study yielded an AUC of 0.604 for the number of dissected lymph nodes, which is relatively low and may impact the reliability of our results. However, another retrospective study also examined ICI efficacy based on the number of dissected lymph nodes, showing a trend where increased lymph node dissection correlated with reduced ICI efficacy, consistent with our findings [23]. Furthermore, their cutoff value for dissected lymph nodes was 16, closely approximating our threshold [23]. This reproducibility strengthens the reliability of our AUC findings and supports the validity of our results.

The clinical implications of this study have the potential to substantially impact both current practices and future research pertaining to the treatment of recurrent NSCLC, and the identification of GC in lymph nodes as a potential biomarker for ICI efficacy offers a new avenue for personalized treatment strategies. These insights could serve as a foundation for developing targeted surgical and immunotherapeutic interventions, thereby enhancing patient outcomes. Specifically, stratifying patients based on the presence of GC to optimize ICI efficacy, and considering lymph node dissection strategies during surgical planning, could help preserve immune function and improve patient outcomes. Furthermore, considering the GC status in resected lymph nodes may potentially aid in determining the eligibility for the use of ICI as adjuvant therapy in the perioperative setting.

Although our study provides additional insights into this area, it is essential to acknowledge its limitations. First, the sample size was relatively small, which may have affected the generalizability of our findings. We did not control for potential confounding variables, such as TPS, comorbidities, and previous treatments' known influence on ICI outcomes, because of the small sample size. Further large-scale studies are required. Second, as this is a retrospective study, it is subjected to inherent limitations such as selection bias. We exclude patients who performed wedge resection since wedge resection does not typically involve lymph node dissection. Prospective validation studies are needed. Third, this study shows that some patients had received chemotherapy as prior treatment. It is possible that prior treatments, such as chemotherapy, could influence the presence of GC. Chemotherapy-induced hypoxia and inflammation in the tumor microenvironment have been reported to negatively affect GC formation, potentially altering the immune dynamics within lymph nodes [16]. This factor should be considered when interpreting the results.

Finally, lymph node assessment was performed by a single pathologist, thus lacking blinding and multidisciplinary review. However, to ensure objectivity, all resected lymph nodes from each specimen were thoroughly evaluated, and immunohistochemical staining was used to complement the pathological assessment. This approach enhances the reliability of our classification and reduces potential bias. Despite these limitations, the strengths of this study lie in its novel focus on GC and lymph node dissection, which provides a robust foundation for future research.

## Conclusions

In conclusion, the findings of this study emphasize the pivotal role of germinal centers in lymph nodes for enhancing the efficacy of ICIs in postoperative recurrent NSCLC. By illuminating how both the presence of GC and the number of dissected lymph nodes may influence therapeutic outcomes, our study opens new avenues for tailored surgical and immunotherapeutic strategies.
